# Association between Urinary Iodine Concentration and Thyroid Nodules in Adults: A Cross-Sectional Study in China

**DOI:** 10.1155/2020/4138657

**Published:** 2020-12-17

**Authors:** Hui Sun, Hanyu Wang, Xiaolan Lian, Chao Liu, Bingyin Shi, Lixin Shi, Nanwei Tong, Shu Wang, Jianping Weng, Jiajun Zhao, Jiaoyue Zhang, Juan Zheng, Xiang Hu, Yunxia Tu, Li Yu, Zhongyan Shan, Weiping Teng, Lulu Chen

**Affiliations:** ^1^Department of Endocrinology, Union Hospital, Tongji Medical College, Huazhong University of Science and Technology, Wuhan 430022, China; ^2^Department of Endocrinology, Beijing Union Medical College Hospital, Beijing 100730, China; ^3^Research Center of Endocrine and Metabolic Diseases, Affiliated Hospital of Integrated Traditional Chinese and Western Medicine, Nanjing University of Chinese Medicine, Nanjing 210028, China; ^4^Department of Endocrinology, The First Affiliated Hospital of Xi'an Jiaotong University, Xi'an 710061, China; ^5^Department of Endocrinology and Metabolism, Affiliated Hospital of Guiyang Medical University, Guiyang 550004, China; ^6^Department of Endocrinology and Metabolism, State Key Laboratory of Biotherapy, West China Hospital, Sichuan University, Chengdu 610041, China; ^7^Department of Endocrinology and Metabolism and the Institute of Endocrinology, Rui-Jin Hospital Affiliated with Shanghai Jiao-Tong University School of Medicine, Shanghai 200025, China; ^8^Division of Life Sciences and Medicine of University of Science and Technology of China, The First Affiliation Hospital of University of Science and Technology of China (Anhui Provincial Hospital), Hefei 230001, China; ^9^Department of Endocrinology, Shandong Provincial Hospital Affiliated with Shandong University, Jinan 250000, China; ^10^Department of Endocrinology and Metabolism and the Institute of Endocrinology, The First Hospital of China Medical University, Shenyang 110001, China

## Abstract

**Background:**

Associations between iodine intake and thyroid nodules (TNs) were not consistent. We aimed to illustrate the relationship between urinary iodine concentration (UIC) and TNs.

**Methods:**

A total of 12,698 participants were enrolled in analysis. All of the participants filled out questionnaires and underwent physical examinations, laboratory tests, and thyroid ultrasonography. UIC, serum thyrotropin (TSH), thyroid peroxidase antibodies (TPOAb), and thyroglobulin antibodies (TgAb) were measured in the central laboratory.

**Results:**

The prevalence of TNs was 16.00%, and the median UIC was 206.1 *μ*g/L. TNs and UIC were negatively related when UIC was less than 527 *μ*g/L (adjusted OR = 0.87; 95% CI, 0.80, 0.94), and the relationship between UIC and TNs was not statistically significant when UIC was greater than 527 *μ*g/L (adjusted OR = 1.25; 95% CI, 0.98, 1.60). In women, UIC was negatively associated with risk for TNs (adjusted OR 0.95; 95% CI, 0.91, 0.99).

**Conclusion:**

The relationship between TNs and UIC differed between men and women. The risk of TNs decreased with the elevation of UIC in men when UIC was lower than 527 *μ*g/L, while UIC and the presence of TNs were negatively correlated in women. In the future, cohort studies or other studies that can explain causality must be conducted to explore the relationship between iodine status and TNs.

## 1. Introduction

The detection of thyroid nodules has become increasingly common with the expanded use of ultrasonographic examinations. The nodules can be found by palpation in about 4%–7% of the population, and approximately 8%–16% of these nodules are malignant [1]. Thyroid nodules cause a number of clinical symptoms, such as vocal hoarseness, compressive symptoms, and dysphagia [2–5], which can harm health. They are associated with many factors, including age, sex, iodine nutrition status, thyroid autoimmune antibodies, and metabolic syndrome [6–8], but of these, iodine nutrition status is one of the most closely related and important factors.

Iodine is an essential trace element necessary for thyroid hormone synthesis and is an indispensable component of the microenvironment that thyroid cells require to thrive. Prior to 1970, China was an iodine-deficient country [9], and this produced various untoward health effects, including goiter, hypothyroidism, hyperthyroidism, abortion, infant mortality, and endemic cretinism [10]. To eliminate iodine deficiency, a mandatory universal salt-iodization program was launched in China in 1996 [9], and the median UIC increased sharply to 330 *μ*g/L in 1997 and then subsequently shifted to 306 *μ*g/L in 1999 [9]. In 2002, the central government revised the national mandate for salt iodization, and the median UIC decreased to 246 *μ*g/L in 2005 [11].

It is generally understood that iodine deficiency increases the risk of thyroid nodules [10, 12]. However, there is little research focusing on the relationship between high iodine intake (UIC ≥ 300 *μ*g/L) and thyroid nodules, and conclusions have not been consistent [12–15]. We performed the present study from 2011 to 2012 to assess iodine nutrition status and to explore the relationship between UIC and thyroid nodules.

## 2. Materials and Methods

### 2.1. Subjects

The subjects who participated in the survey were chosen from 10 cities in eastern and central China in 2011–2012. These were Shenyang, Beijing, Jinan, Xi'an, Chengdu, Nanjing, Shanghai, Wuhan, Guiyang, and Guangzhou. We used cluster random sampling in the survey. In each city, we chose an urban area randomly as the basic sampling unit, and one community was randomly selected from the selected urban area. In the selected community, housing estates were numbered, and one or two housing estates were selected. Then, we numbered the buildings belonging to the housing estates and randomly selected the building to be investigated first. If the number of subjects did not meet the requirements of sample size in the first housing estate, a second housing estate was then randomly selected. We started from the first household of the first building selected, and if the residents met the inclusion criteria and were willing to participate in our survey, they were registered. The number of subjects registered was 20 percent greater than the actual number of people surveyed. In accordance with the principle of cluster sampling, only the registered persons could participate in the survey, avoiding a tendency to seek medical treatment. Finally, about 1500 individuals were selected and stratified by age and sex in each city, and the age and sex composition of each city was then determined by referring to China's 2008 national census data. Eventually, a total of 15,008 individuals were recruited to our study. The inclusion criteria were as follows: Han nationality, aged 15 years or older, and residence in the specified community for at least 10 years. People were not eligible if they had received iodine drugs or contrast agents within the past 3 months; were pregnant or lactating; suffered from adrenocortical hypofunction, hepatic or renal dysplasia, or other serious systemic or chronic wasting disease; or were receiving any drugs capable of affecting thyroid function, such as glucocorticoids, dopamine, or dobutamine.

The research protocols were reviewed and approved by the medical ethics committee of China Medical University (serial number, IRB [2008]34). Before we collected data, all subjects provided written informed consent.

### 2.2. Measurements

Only investigators who had passed the performance assessment could administer the survey. For each participant, personal information was collected via a questionnaire that included name, sex, age, address, contact information, childbearing history, educational level, smoking status, type of salt used, family and personal history of thyroid disease, and medication status. We defined smoking status as smokers and nonsmokers; “smokers” smoked one or more cigarettes per day, continuously, or cumulatively smoking for six months or more [16].

Doctors at the local hospital who had passed the training provided participants with a physical examination and took detailed anthropometric measurements. Weight and height were measured with participants wearing light clothing and no shoes; BMI was calculated as weight in kilograms divided by squared height in meters, and waist circumference was measured at a level midway between the lowest rib and the iliac crest.

Thyroid ultrasonographic evaluations were performed using a portable instrument (LOGIQ a50, 7.5 MHz; GE Healthcare) by experienced physicians who had received three days centralized training in the First Hospital of China Medical University. The instruments used for this research were assessed and standardized in a timely fashion, and data were entered by two individuals and evaluated as to their consistency. Interviewers and physicians were overseen by members of the steering committee. The thyroid ultrasound report included the size of thyroid, the internal echo of thyroid, and the number, size, and internal echo of the thyroid nodules.

Urine and blood samples were collected from every subject in the morning after an overnight fast. UIC was detected by the ammonium persulfate method based on the Sandell-Kolthoff reaction (UV-1600 spectrophotometer, Ruili Analytical Instrument Group Co., Ltd.; China) at the Key Laboratory of Endocrine Diseases in Liaoning Province, Department of Endocrinology and Metabolism, and the Institute of Endocrinology, The First Hospital of China Medical University. The quality of the measurements was controlled against the certified reference material (GBW09109) from the Center for Disease Control (CDC) in China. The reference range of the certified reference material from CDC in China was 138 ± 10 *μ*g/L (mean ± standard deviation), and the result measured by the key laboratory was 134.3 ± 6.2 *μ*g/L (mean ± standard deviation). The inter- and intra-assay coefficients of variation for UIC were 4%–6% and 3%–4% at 66 *μ*g/L and 3%–6% and 2%–5% at 230 *μ*g/L, respectively.

Serum concentrations of thyrotropin (TSH), thyroid peroxidase antibodies (TPOAb), and thyroglobulin antibodies (TgAb) were measured using an electrochemiluminescence immunoassay (Cobas 601 analyzer, Roche Diagnostics). The reference ranges provided by the test kit manufacturer were 0.27–4.2 mIU/L for TSH, 0–34 IU/mL for TPOAb, and 0–115 IU/mL for TgAb. The sensitivity of serum TSH was 0.002 mIU/L. The interassay CV values for serum TSH, TPOAb, and TgAb were 1.9%–9.5%, and the intra-assay CV values were 1.1%–6.3%.

### 2.3. Statistical Analysis

**Figure 1:**
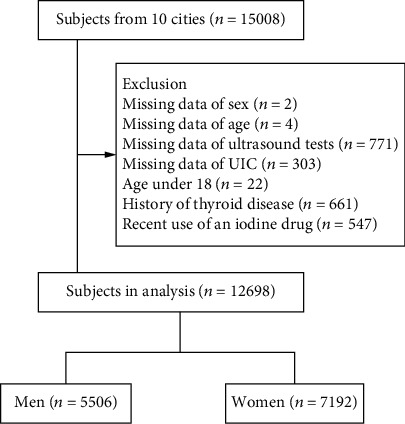
Flowchart of the inclusion and exclusion of participants.

Our study initially involved 15,008 participants, of whom 2310 were excluded due to missing data regarding age, sex, ultrasonographic examinations, and UIC; age under 18; a history of thyroid diseases; or recent use of an iodine-containing or modulating drug. Thus, 12,698 individuals were eligible for analysis (Figure 1).

All continuous variables conformed to a nonnormal distribution and were therefore presented as medians and interquartile ranges. The categorical variables were presented as counts and percentages. Comparisons of these variables between two groups were performed using the Mann–Whitney (for nonnormally distributed data) for continuous variables and *χ*2 tests for categorical variables. We explored the relationship between UIC and thyroid nodules by smooth curve fitting after adjustment for potential confounders. Then, using a segmented regression model, we used the LRT test (likelihood-ratio test) to analyze the threshold effect between UIC and thyroid nodules. Multivariate logistic analysis was used to evaluate the relationships between UIC and thyroid nodules. Values of *p* < 0.05 were considered statistically significant, and statistical analysis was performed using Empower (R) (http://www.empowerstats.com, X&Y solutions, Inc. Boston MA) and R (http://www.R-project.org).

## 3. Results

### 3.1. General Information of Subjects

As shown in Table 1, there were 5506 (43.36%) men and 7192 (56.64%) women enrolled in the present study. The prevalence of thyroid nodules was 16.00% for all adults, and women had a much higher prevalence of thyroid nodules than men (17.74% vs. 13.73%, *p* < 0.001). The median age was 44 years, and the median UIC value was 206.09 *μ*g/L. The median values for BMI and waist circumference were 23.66 kg/m^2^ and 80.00 cm, respectively. Iodized salt was used by 98.52% of our subjects. We used univariate analysis and found that elderly, women, obese subjects, nonsmokers, subjects who had less education, and those with high blood pressure were more likely to suffer from thyroid nodules (all *p* < 0.05).

### 3.2. Associations between UIC and Thyroid Nodules

**Figure 2:**
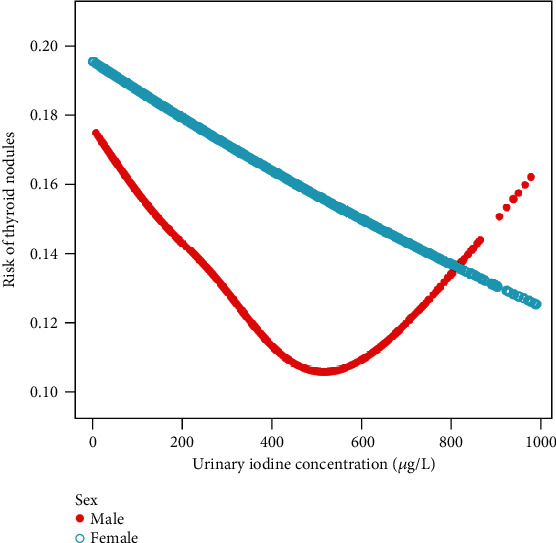
The relationship between UIC and risk of thyroid nodules in adults stratified by sex. Similar U-shaped relationship between UIC and thyroid nodules was observed in men, but linear relationship was observed in women after adjusting for age, educational status, smoking status, type of salt used, family history of thyroid diseases, systolic pressure, body mass index, and waist circumference by smooth curve fitting.

A similar U-shaped relationship between UIC and risk for thyroid nodules was observed in males after adjusting for age, educational status, smoking status, type of salt used, family history of thyroid diseases, systolic pressure, body mass index, and waist circumference after meeting the filter criteria, while the same trend was not observed for females (Figure 2). A turning point of UIC (527 *μ*g/L) was found by using a segmentation regression model between UIC and risk for thyroid nodules in males. A trend for a positive relationship was also uncovered between UIC and thyroid nodules when UIC was greater than 527 *μ*g/L (adjusted OR = 1.25; 95% CI, 0.98, 1.60; *p* = 0.076). However, we observed a negative association between UIC and thyroid nodules when UIC was lower than 527 *μ*g/L (adjusted OR = 0.87; 95% CI, 0.80, 0.94; *p* < 0.001), and using LRT, we achieved *p* of 0.018, indicating that a nonlinear relationship existed between UIC and thyroid nodules. However, UIC was negatively correlated with the risk for thyroid nodules in females (OR 0.95; 95% CI, 0.91, 0.99; *p* = 0.023) (Table 2).

### 3.3. Joint Effects between Different Factors and UIC on the Risk for Thyroid Nodules

In general, the associations between UIC and thyroid nodules were consistent across different risk-strata subgroups as defined by age, sex, TSH, BMI, TPOAb (+), and TgAb (+), and we found no significant interaction (all *p* interaction > 0.05) (Table 3).

## 4. Discussion

In our study, we found that UIC was negatively correlated with risk of thyroid nodules when UIC fell within the range of 0–527 *μ*g/L and was not correlated with thyroid nodules when it was above 527 *μ*g/L. However, with respect to women, iodine intake was negatively correlated with thyroid nodules. Furthermore, the relationship between UIC and thyroid nodules was consistent across different subgroups.

Many studies have shown that subjects with low UIC (<100 *μ*g/L) exhibited an increased risk for thyroid nodules [10, 12, 17, 18]. A median UIC between 100 and 300 *μ*g/L was considered to constitute adequate iodine intake, and more-than-adequate iodine intake in the overall population based on WHO criteria, and was considered to pose few risks of thyroid nodules [19]. Our questions thus focused on the associations between excessive iodine intake (median UIC ≥ 300 *μ*g/L) and thyroid nodules. Chen et al. found that UIC ≥ 300 *μ*g/L did not increase the risk of thyroid nodules in a study conducted in Zhejiang Province (a coastal region) that included 9412 adults with a median UIC of 100–199 *μ*g/L [12], while Sun et al. found that UIC ≥ 300 *μ*g/L increased risk for thyroid nodules [13]. In the latter study, the investigators selected three areas with completely different iodine nutrition statuses and compared the prevalence of thyroid nodules according to the classification of regions [13]. Kim et al. found that UIC ≥ 2500 *μ*g/L was associated with thyroid cancer but that UIC between 300 and 2500 *μ*g/L did not increase risk. Kim et al. conducted the study in Korea, which was an iodine-replete area, and the iodine status there was historically much higher than in China [15]. Different statistical methods, different cut-off values for iodine nutrition status within different investigations, and regional differences in iodine intake, eating habits as well as ethnicity are possible explanations for these inconsistent results. When iodine is deficient, TSH release is augmented to maximize the uptake of iodine, and TSH subsequently induces hypertrophy and hyperplasia of the thyroid gland. Thyroid nodules then develop after a certain period of time, with many nodules derived from a somatic mutation and of monoclonal origin. Iodine deficiency is, in fact, a growth promoter of these mutations [20, 21]. While the mechanism(s) underlying the association between high levels of iodine intake and thyroid nodules remains unclear. The high urinary iodine concentrations may promote thyrocyte proliferation at an increased rate, allowing the dividing cells to accumulate more genetic alterations and making the thyrocytes vulnerable to mutagens [22–25]. But iodine can also exhibit antiproliferative properties [26].

We found that the relationship between UIC and thyroid nodules was inconsistent in males and females. In men, deficient iodine intake was not optimal for thyroid nodules. When UIC was higher than 527 *μ*g/L, there was a positive trend between UIC and the risk of thyroid nodules, although it was not statistically significant. However, for women, it appeared that the higher the level of UIC, the lower the risk for thyroid nodules, which is intriguing, because we know that the occurrence of thyroid nodules is more frequent in women. The reasons for this apparent dichotomy are complex and poorly understood, which may relate to the impact of estrogens and genetic factors [27–29]. Estrogen receptors alpha (ER-*α*) and beta (ER-*β*) were known to expressed on thyroid follicular cells, with ER-*α* inducing proliferation and ER-*β* decreasing proliferation and promoting apoptosis [30]. When estrogen level was higher, the balance between ER-*α* and ER-*β* would be affected, which was beneficial to proliferation. This response was more pronounced in females [31]. And estrogen can inhibit the expression of sodium iodide symporter (NIS), which played an important role in iodine transport of thyroid gland [32]. Gender differences between UIC and thyroid nodules maybe the result of complex interactions of sex hormones, iodine, and thyrocyte, which need to be explored further.

Our study has some limitations. First, spot UIC is not stable because it is influenced by food intake and the extent of hydration [33]. However, variations generally even out in large numbers of samples [10, 33], and UIC is a well-accepted indicator of iodine status that is used for assessment at the population level [10]. Moreover, the cross-sectional nature of the present survey also indicated that we were unable to determine the mechanisms underlying the observed phenomena. Additional cohort studies—or other studies that can explain a causal relationship—need to be conducted.

## 5. Conclusions

In conclusion, the effect of iodine on thyroid nodules might differ between men and women. For men, UIC and thyroid nodules were negatively related when UIC was lower than 527 *μ*g/L, and they had a positive trend when UIC was higher than 527 *μ*g/L although it was not significant statistically. For women, it appeared that higher UIC was negatively associated with the presence of thyroid nodules. Sex differences, therefore, might be taken into account in the use of scientific iodine supplements. In the future, additional studies that can explain causality should be conducted to allow investigators to explore the relationships between iodine status and thyroid nodules.

## Figures and Tables

**Table 1:** Distribution of characteristics among subjects with and without thyroid nodules^a^.

Variables	Total subjects	Nonnodules	Nodules	*p*
*N*	12698	10666	2032	
Sex, *n* (%)				<0.001
Male	5506 (43.36)	4750 (44.53)	756 (37.20)	
Female	7192 (56.64)	5916 (55.47)	1276 (62.80)	
Age, years	44.00 (33.00–56.00)	42.00 (32.00–53.00)	54.00 (44.00–65.00)	<0.001
Menopause, *n* (%)				<0.001
No	4428 (62.93)	3907 (67.54)	521 (41.65)	
Yes	2608 (37.07)	1878 (32.46)	730 (58.35)	
Smoking status, *n* (%)				0.001
Nonsmokers	9554 (75.24)	7967 (74.70)	1587 (78.10)	
Smokers	3144 (24.76)	2699 (25.30)	445 (21.90)	
Educational status, *n* (%)				<0.001
Absent from education	244 (1.93)	171 (1.61)	73 (3.62)	
Primary and junior high school	3755 (29.78)	3012 (28.43)	743 (36.87)	
Senior high school	3694 (29.29)	3125 (29.50)	569 (28.24)	
College and above	4917 (38.99)	4287 (40.46)	630 (31.27)	
Type of salt used, *n* (%)				0.707
Iodized	12485 (98.52)	10489 (98.51)	1996 (98.62)	
Noniodized	187 (1.48)	159 (1.49)	28 (1.38)	
Family history of thyroid diseases, *n* (%)				0.189
No	11950 (94.70)	10053 (94.81)	1897 (94.10)	
Yes	669 (5.30)	550 (5.19)	119 (5.90)	
BMI, kg/m^2^	23.66 (21.34–26.04)	23.45 (21.18–25.91)	24.53 (22.31–26.72)	<0.001
Waist circumference, cm	80.00 (74.00–88.00)	80.00 (73.00–87.00)	83.00 (76.00–90.00)	<0.001
Systolic pressure, mmHg	120.00 (110.00–130.00)	120.00 (109.00–130.00)	125.00 (110.00–140.00)	<0.001
Diastolic pressure, mmHg	78.00 (70.00–84.00)	78.00 (70.00–82.00)	80.00 (70.00–86.00)	<0.001
TSH, mIU/L	2.43 (1.67–3.54)	2.46 (1.70–3.56)	2.29 (1.52–3.40)	<0.001
TPOAb, IU/mL	8.17 (5.55–11.92)	8.18 (5.55–11.98)	8.03 (5.58–11.57)	0.274
TgAb, IU/mL	10.00 (10.00–11.74)	10.00 (10.00–11.77)	10.00 (10.00–11.54)	0.181
UIC, *μ*g/L	206.09 (137.85–301.01)	210.60 (140.87–307.41)	186.31 (123.59–270.85)	<0.001

^a^Data are described as median with interquartile range for continuous variables or as counts with percentages for categorical variables. BMI: body mass index; TSH: serum thyrotropin; TPOAb: thyroid peroxidase antibodies; TgAb: thyroglobulin antibodies; UIC: urinary iodine concentration.

**Table 2:** Threshold effect analysis for the relationship between UIC and the risk of thyroid nodules^a^.

Models	Male		Female	
	Risk of TNs adjusted OR (95% CI)	*p*	Risk of TNs adjusted OR (95% CI)	*p*
Model I				
One-line slope	0.92 (0.86, 0.97)	0.006	0.95 (0.91, 0.99)	0.023
Model II				
Turning point (K)	5.27		4.79	
<K slope 1	0.87 (0.80, 0.94)	<0.001	0.92 (0.86, 0.98)	0.008
>K slope 2	1.25 (0.98, 1.60)	0.076	1.06 (0.91, 1.23)	0.478
Slope 2–slope 1	1.44 (1.08, 1.92)	0.013	1.15 (0.95, 1.39)	0.150
Predicted at K	-2.50 (-2.75, -2.25)		-2.01 (-2.19, -1.83)	
LRT test	0.018		0.156	

^a^Adjusted for age, educational status, smoking status, type of salt used, family history of thyroid diseases, systolic pressure, body mass index, and waist circumference. Model I, linear analysis; Model II, nonlinear analysis; LRT test: likelihood ratio test; TNs: thyroid nodules. The value of OR corresponded to per 100 units change of urinary iodine concentration.

**Table 3:** Associations between each 100 *μ*g/L increase in UIC and thyroid nodules in subgroups^a^.

Variables	*N*	OR (95% CI)	*p*	*p* interaction
Age, years				0.579
<30	2281	0.89 (0.78, 1.01)	0.075	
30 to <40	2856	0.89 (0.81, 0.99)	0.025	
40 to <50	2788	0.93 (0.86, 1.01)	0.097	
50 to <60	2418	0.96 (0.90, 1.03)	0.309	
60 to <70	1293	0.88 (0.80, 0.97)	0.012	
≥70	1047	0.96 (0.87, 1.06)	0.406	
BMI^b^, kg/m^2^				0.798
<18.5	547	0.96 (0.78, 1.18)	0.681	
18.5 to <24	6177	0.89 (0.84, 0.94)	<0.001	
24 to <28	4350	0.88 (0.83, 0.94)	<0.001	
≥28	1450	0.94 (0.85, 1.04)	0.204	
TSH^c^, mIU/L				0.147
<0.27	152	0.87 (0.61, 1.23)	0.432	
0.27 to <4.2	10278	0.90 (0.87, 0.94)	<0.001	
≥4.2	2023	0.81 (0.73, 0.90)	<0.001	
TPOAb (+)^d^, IU/mL				0.023
No	11366	0.90 (0.87, 0.94)	<0.001	
Yes	436	0.67 (0.51, 0.88)	0.005	
TgAb (+)^e^, IU/mL				0.470
No	11213	0.90 (0.86, 0.93)	<0.001	
Yes	354	0.95 (0.75, 1.20)	0.663	

^a^Adjusted for age, sex, educational status, smoking status, type of salt used, family history of thyroid diseases, systolic pressure, body mass index, and waist circumference. ^b^BMI category according to the Ministry of Health of the People's Republic of China classification: underweight (<18.5 kg/m^2^), normal weight (18.5 to <24 kg/m^2^), overweight (24 to <28 kg/m^2^), and obese (≥28 kg/m^2^). ^c^TSH category: below reference range (<0.27 mIU/L), reference range (0.27 to <4.2 mIU/L), and above reference range (>4.2 mIU/L). ^d^TPOAb (+): no (TPOAb ≤ 34 IU/mL) and yes (TPOAb > 34 IU/mL). ^e^TgAb (+): no (TgAb ≤ 115 IU/mL) and yes (TgAb > 115 IU/mL).

## Data Availability

The datasets generated and/or analyzed during the current study are available from the corresponding author on reasonable request.

## Abbreviations

UIC: Urinary iodine concentration
TNs: Thyroid nodules
TSH: Serum thyrotropin
TPOAb: Thyroid peroxidase antibodies
TgAb: Thyroglobulin antibodies
LRT test: Likelihood-ratio test.
